# Sequencing and Characterization of Novel PII Signaling Protein Gene in Microalga *Haematococcus pluvialis*

**DOI:** 10.3390/md15100304

**Published:** 2017-10-11

**Authors:** Ruijuan Ma, Yan Li, Yinghua Lu

**Affiliations:** 1 Department of Chemical and Biochemical Engineering, College of Chemistry and Chemical Engineering, Xiamen University, Xiamen 361005, China; ruijuanma@hotmail.com; 2 Algae Biotechnology Laboratory, School of Agriculture and Food Sciences, The University of Queensland, Brisbane 4072, Queensland, Australia

**Keywords:** *Haematococcus pluvialis*, PII signaling protein, nitrogen starvation, gene cloning, mRNA expression

## Abstract

The PII signaling protein is a key protein for controlling nitrogen assimilatory reactions in most organisms, but little information is reported on PII proteins of green microalga *Haematococcus pluvialis*. Since *H. pluvialis* cells can produce a large amount of astaxanthin upon nitrogen starvation, its PII protein may represent an important factor on elevated production of *Haematococcus* astaxanthin. This study identified and isolated the coding gene (Hp*GLB1*) from this microalga. The full-length of Hp*GLB1* was 1222 bp, including 621 bp coding sequence (CDS), 103 bp 5′ untranslated region (5′ UTR), and 498 bp 3′ untranslated region (3′ UTR). The CDS could encode a protein with 206 amino acids (HpPII). Its calculated molecular weight (Mw) was 22.4 kDa and the theoretical isoelectric point was 9.53. When *H. pluvialis* cells were exposed to nitrogen starvation, the Hp*GLB1* expression was increased 2.46 times in 48 h, concomitant with the raise of astaxanthin content. This study also used phylogenetic analysis to prove that HpPII was homogeneous to the PII proteins of other green microalgae. The results formed a fundamental basis for the future study on HpPII, for its potential physiological function in *Haematococcus* astaxanthin biosysthesis.

## 1. Introduction

Inorganic nitrogen acts as an important nutrient in autotrophic microalgae cultivation. It is a limiting factor for cell growth. While under a nitrogen-depleted condition, microalgae will transfer the energy from cell division/growth to produce more secondary metabolites like carotenoids [1,2] and lipids [3], which have widespread commercial value. Therefore, nitrogen regulation is an important approach for microalgae cultivation. 

It is believed that nitrogen metabolism is regulated by multiple signal regulators and linked to carbon flux [4]. In most organisms, PII proteins act as the central signal-integrating molecules for controlling nitrogen and/or carbon metabolism, along with some effector molecules (e.g., ATP and 2-oxoglutarate) [5,6,7]. These molecules bind to the intercommunicating sites of the trimeric PII proteins, forming different PII conformations to control a variety of enzymes, transcription factors, and membrane transport proteins [8,9,10]. Although PII proteins have highly conserved structures, they interact with various target metabolisms in different organisms. In cyanobacteria and plants, for example, PII proteins can control the activity of *N*-acetyl-l-glutamate kinase (NAGK), and then regulate the metabolism of glutamate towards arginine and polyamines [5,11]. In plant chloroplast and bacteria, acetyl-CoA carboxylases, catalysing the committed and rate-limiting step in fatty acid biosynthesis, are regulated by PII proteins [12,13]. It is believed that PII proteins may also play some uncharacterized roles in plant cells [5,14]. 

Compared to the study on plants and bacteria, research of PII proteins in microalgae is still in the primary stage. To date, the PII proteins have only been reported in two green microalgae *Chlamydomonas reinhardtii* [15] and *Chlorella variabilis* [14]. It has been reported that PII proteins originate from cyanobacteria and conserve in the evolution of higher plants [5]. Since green algae are in the phylogenetic lineage between cyanobacterial ancestor and higher plants [14], phylogenetic analysis seems to be an efficient approach to verify the newly identified PII protein of the target microalgae, aligned with some reported PII proteins in plants and cyanobacteria. Given the importance of nitrogen metabolism in microalgae, it is essential to characterize more PII proteins and also verify relevant metabolic functions.

The green microalga *Haematococcus pluvialis* is well known due to its extreme capability of producing a large amount of powerful antioxidant-astaxanthin [16,17,18]. Driven by their nutrition condition, *H. pluvialis* cells have two different physiological traits: (1) in favorable conditions they are in a green motile stage; (2) under stress conditions (especially nitrogen depletion) the green cells will transform into a reddish non-motile resting stage, coupled with astaxanthin accumulation [19,20,21]. Several key genes related to astaxanthin biosynthesis and stress responding have been cloned and characterized, such as *pds* [22], *CYP97C* [23], *MnSOD* [24], and *TR1* [25]. Despite many reports on astaxanthin accumulation of *H. pluvialis* upon nitrogen depletion [26,27,28], there is no information of its PII protein and associated genetic transcription information on this unicellular microalga. 

In this study, we cloned the full length of the PII signaling protein gene on *H. pluvialis*, analyzed the phylogenetic relationship and structure of this protein, and also investigated its time course-dependent transcriptional regulation. It is anticipated that the results will provide fundamental knowledge on the PII protein of *H. pluvialis*, and also highlight the importance of its potential regulation/interactions for astaxanthin biosynthesis.

## 2. Results

### 2.1. Cloning and Characterization of HpGLB1

**Figure 1:**
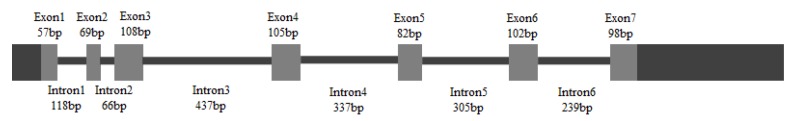
The gene structure of Hp*GLB1.* Grey boxes and black solid lines represent exons and introns, respectively. Black boxes represent 5′ and 3′ UTRs.

A 148 bp cDNA fragment of Hp*GLB1* was obtained from *H. pluvialis* cells by RT-PCR in this study. This fragment was homologous to the *GLB1* of *Chlamydomonas reinhardtii* and *Chlorella variabilis*. The results showed that 5′-RACE PCR generated a 568 bp fragment, and 3′-RACE PCR generated a 702 bp fragment. Alignment assay indicated that the complete cDNA sequence of Hp*GLB1* was 1222 bp, including 621 bp coding sequence (CDS), 103 bp 5′ untranslated region (5′ UTR), and 498 bp 3′ untranslated region (3′ UTR). The length of the open reading frame of Hp*GLB1* in genomic DNA was 2123 bp, containing 7 exons and 6 introns (Figure 1). The length of introns varied from 66 bp to 437 bp. The sequence of Hp*GLB1* has been submitted to NCBI GenBank (Accession number: KT696441).

### 2.2. Characterization of HpPII Protein

**Figure 2:**
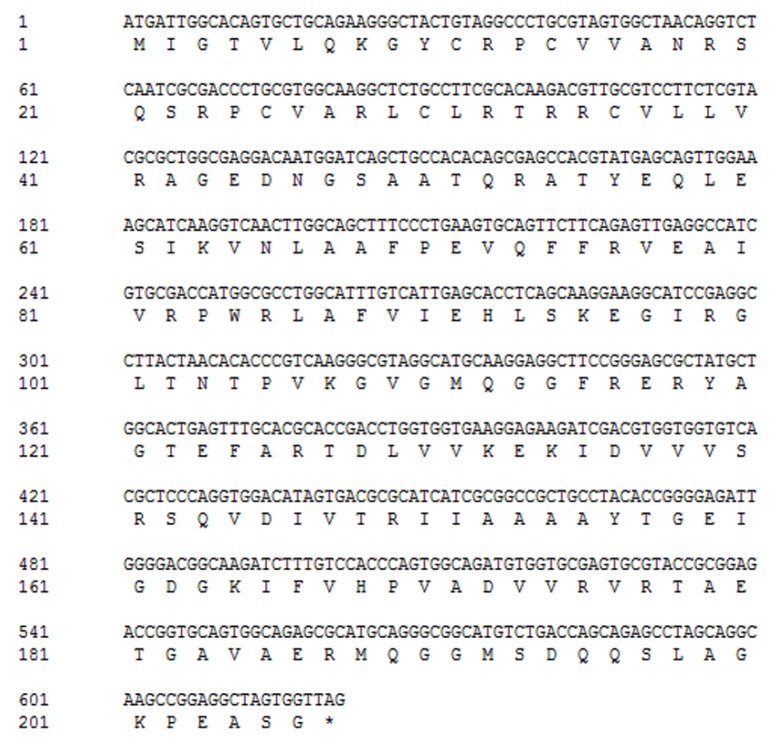
Nucleotide and deduced amino acid sequence of Hp*GLB1*. The asterisk represents the stop codon.

**Figure 3:**
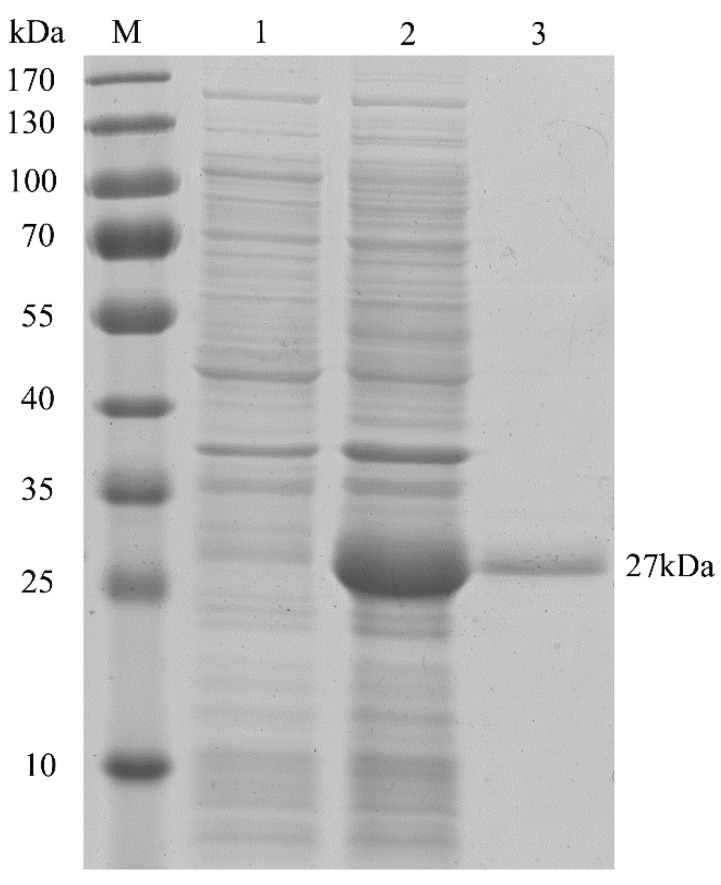
Sodium dodecyl sulfate-polyacrylamide gel electrophoresis (SDS-PAGE) analysis of HpPII in *E. coli* BL21 (DE3). M: protein marker; lane 1: total proteins extracted from uninduced pET28a-HpG*LB1* (no HpPII expression, control); lane 2: total proteins extracted from induced pET28a-HpG*LB1* (with HpPII expression); lane 3: purified HpPII protein.

Encoded by Hp*GLB1* cDNA, the deduced full-length HpPII protein consisted of 206 amino acid residues (Figure 2). According to the Computer pI/Mw Tool, the calculated values of pI and Mw were 9.53 and 22.4 kDa, respectively. However, the molecular mass was approximately 27 kDa after HpPII was expressed and purified in *E. coli* BL21 (DE3) (Figure 3). This was the sum of the calculated Mw of HpPII (22.4 kDa) and N-terminal tag (4 kDa), and the latter was from the plasmid pET28a used for purification.

### 2.3. Multiple Sequence Alignment and Structural Prediction

**Figure 4:**
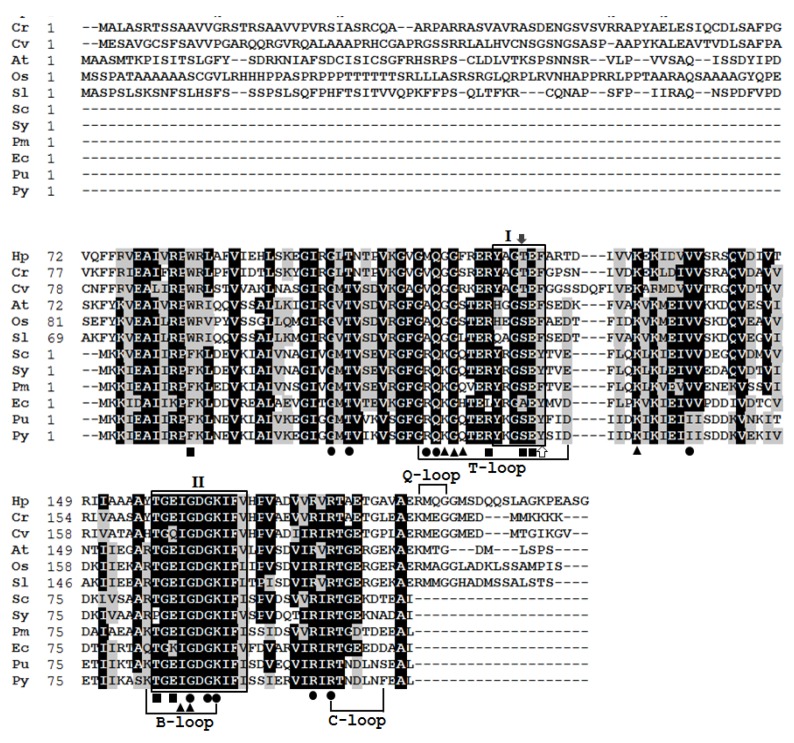
Alignment of the amino acid sequences of PII proteins among different organisms: *Haematococcus pluvialis* (Hp; AOO85416), *Chlamydomonas reinhardtii* (Cr; EDO96407.1), *Chlorella variabilis* (Cv; AHW46897.1), *Arabidopsis thaliana* (At; AAC78333.1), *Oryza sativa* (Os; NP_001054562.1), *Solanum lycopersicum* (Sl; NP_001234506.1), *Synechocystis* sp. PCC 6803 (Sc; WP_010873156.1), *Synechococcus* sp. (Sy; AAA27312.1), *Prochlorococcus marinus* (Pm; WP_036930683.1), *Escherichia coli* (Ec; CDZ21367.1), *Porphyra umbilicalis* (Pu; AFC39923.1), *Pyropia yezoensis* (Py; YP_536935.1). Residues in black represent >60% identity of aligned PII proteins. Amino acids shaded with grey display similar residues. Box I and box II are PII signature patterns I and II. The white arrow indicates the residue of the corresponding uridylylated threonyl-residue in proteobacteria. The black arrow indicates the residue of corresponding phosphorylated serine-residue in cyanobacteria. Black dots, squares, and triangles show the ATP-, NAGK-, and 2KG-binding residues, respectively.

The derived HpPII protein was aligned with the sequences of representative PII protein from green algae, plants, cyanobacteria, and bacteria (Figure 4). Like the PII proteins of *C. reinhardtii* and *C. variabilis*, HpPII had N- and C-terminal extensions, which did not exist in prokaryotes. Furthermore, the sequence from residue 74 to 186 encompassed homology to the entire 112 residues of prokaryotic PII proteins. Within the homology region, the identities of HpPII were aligned up to 78% and 63% with microalgae *C. reinhardtii* and *C. variabilis*, respectively. HpPII also had a high similarity with the PII proteins of plants, cyanobacterial, bacterial and red algal, such as: *Arabidopsis thaliana* (55%), *Oryza sativa* (52%), *Solanum lycopersicum* (54%), *Synechocystis* sp. PCC 6803 (50%), *Synechococcus* sp. (47%), *Prochlorococcus marinus* (47%), *Escherichia coli* (45%), *Porphyra umbilicalis* (43%), and *Pyropia yezoensis* (41%).

Two signature patterns (I and II) of extremely high similarity have been proposed at the PROSITE (PS00496 and PS00638) (Figure 4). *Escherichia coli* of proteobacteria has tyrosyl-residue (Tyr-51) in signature pattern I, which is posttranslational modified by uridylylation. However, in HpPII and PII proteins of green algae *C. reinhardtii*, *C. variabilis*, and higher plants, the Tyr-51 residue is substituted by phenylalanyl-residue (Figure 4). Although PII proteins of *Synechococcus* comprise Tyr-51 residue, they are not uridylylated, due to being modified by phosphorylation at seryl-residue (Ser-49) [29]. Nevertheless, the corresponding Ser-49 residue is replaced by threoninyl-residue in HpPII and PII proteins of green algae *C. reinhardtii* and *C. variabilis* (Figure 4). Similar to the other PII proteins, HpPII has T loop, B loop, and C loop (Figure 4). In addition, HpPII also has a consensus sequence of Q loop (R/K, M, Q, G) structure in C-terminal extension (Figure 4), which is comparable to the PII proteins of *C. reinhardtii*, *C. variabilis*, and higher plants. They are known to constitute binding sites of metabolite effectors [8]. These binding residues are highlighted in Figure 4.

### 2.4. Phylogenetic Analysis

**Figure 5:**
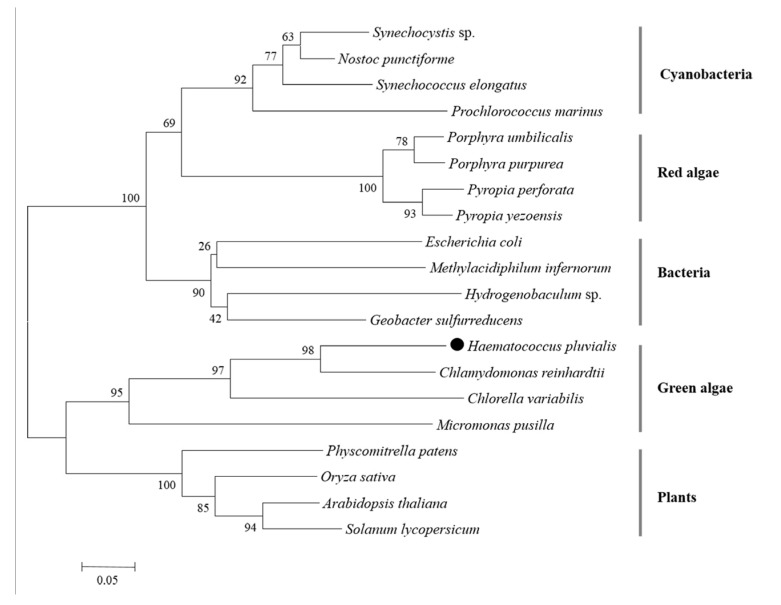
Phylogenetic tree of PII proteins in different organisms (the black dot represents the derived HpPII in this study). The phylogenetic tree was constructed by the Neighbor-joining method, and the numbers above the nodes represent the bootstrap values.

A Neighbor-Joining tree was generated by PII proteins of microalgae, plants, and bacteria via MEGA 6.06 software. There were five clusters: cyanobacteria, red algae, bacteria, green algae, and plants (Figure 5). HpPII protein was grouped with the PII proteins of Chlorophyta algae (*C. reinhardtii*, *C. variabilis*, *Micromonas pusilla*).

### 2.5. Transcription Analysis of HpGLB1 under Nitrogen Deprivation Condition

**Figure 6:**
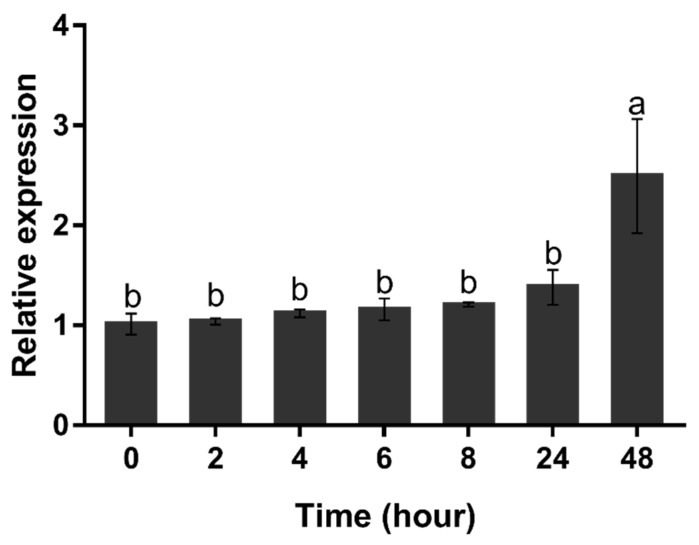
Transcription level of Hp*GLB1* under nitrogen deprivation condition. Data in this figure are the mean values of biological triplicates, and the error bar indicates their standard error (SE). Statistical significance differences are represented by different letters at *p* < 0.05. The *H. pluvialis* cells (of 5 × 10^5^ cells mL^−1^) were resuspended in BBM-N medium at 0 h (control). The transcription level of Hp*GLB1* were measured at 0, 2, 4, 6, 8, 24, 48 h by qRT-PCR. The 18S ribosomal RNA gene was used as the reference gene, and the values were normalized to the transcription level of the control.

When *H. pluvialis* cells were exposed to nitrogen starvation, the expression level of Hp*GLB1* mRNA hardly changed from 0 to 24 h (*p* > 0.05) (Figure 6). However, its transcription level increased significantly at 48 h (*p* < 0.05).

### 2.6. Astaxanthin Accumulation Pattern under Nitrogen Deprivation Condition

**Figure 7:**
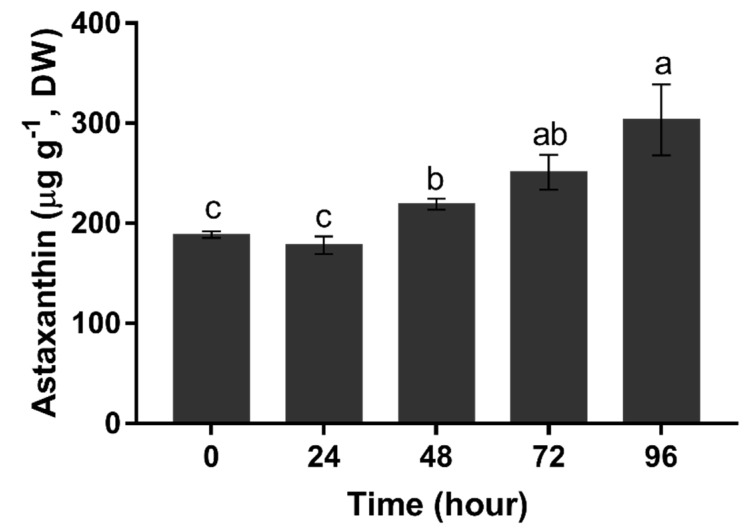
Total astaxanthin content of *H. pluvialis* upon nitrogen starvation. Data in this figure are the mean values of biological triplicates, and the error bar indicates their standard error (SE). Statistical significance differences are represented by different letters at *p* < 0.05.

The content of astaxanthin was maintained below 200 μg g^−1^ in the first 24 h (*p* > 0.05). It started to increase at 48 h, and then continuously accelerated from 48 h (219.02 μg g^−1^) to 96 h (303.25 μg g^−1^) (Figure 7).

## 3. Discussion

This study is the first to isolate and identify a novel gene of PII protein (Hp*GLB1*) on green algae *H. pluvialis*. The complete cDNA sequence of Hp*GLB1* was 1222 bp, including 621 bp of CDS, 103 bp 5′UTR, and 498 bp 3′UTR (Figure 1). The open reading frame of Hp*GLB1* in genomic DNA contained 7 exons and 6 introns, which is the same as for green microalga *C. variabilis* [14]. It is reported that PII proteins are chloroplast genome-encoded in red algae [6] but in green microalgae they are encoded by nuclear genome [14]. Since *H. pluvialis* is a green alga and has the same exons and introns distribution of *GLB1* as that of *C. variabilis*, the HpPII is deemed nuclear-encoded in *H. pluvialis* as well.

The coding sequence (CDS) of Hp*GLB1* gene encoded a protein of 207 amino acids with 22.4 kDa of calculated weight (Figure 2). When HpPII was expressed in *E. coli* BL21 (DE3), the purified protein Mw was about 27 kDa (Figure 3). Since there was 4 kDa of N-terminal tag, the remaining Mw of protein was similar to the calculated weight of HpPII. In this study, the length of Hp*GLB1* CDS and the Mw of HpPII were similar to the PII proteins of green microalgae *C. reinhardtii* (615 bp, 22 kDa) [15] and *C. variabilis* (630 bp, 22 kDa) [14]. These results indicate that these PII proteins are conserved in green microalgae.

HpPII had a high similarity in the segment of 75 and 185 amino acids to the eukaryotic PII proteins (Figure 4). This was consistent with the phylogenetic analysis of HpPII being in the same cluster with three other green algal PII proteins from *C. reinhardtii*, *C. varLiabilis*, and *M. pusilla*. Since the specific Tyr-51 (uridylylated site) was substituted with phenylalanyl-residue in the signature pattern I region of HpPII (Figure 4), it is indicated that HpPII is not modified by uridylylation. The absence of modification by uridylylation is similar to the literature reports on green algae *C. reinhardtii* and *C. variabilis* [14,15]. The Ser-49 was replaced by a threonyl residue in PII protein (Figure 4). This result is the same as PII protein of *C. reinhardtii*, which has been verified not being modified by phosphorylation [15]. Therefore, it is deduced that HpPII is also not be regulated by phosphorylation.

Different from prokaryotic PII proteins, HpPII had extra N- and C-terminal extensions. As highlighted in green algae and plants, they would represent some additional functions [15,30]. A consensus sequence of Q loop (R/K, M, Q, G) was found in the C-terminal extension of HpPII (Figure 4). Q loop was also observed in PII proteins of green microalgae *C. reinhardtii* and *C. variabilis* [14,15]. As reported in *C. reinhardtii*, Q loop was deemed to play a role in nitrogen assimilation, binding glutamine molecule and forming glutamine-dependent complex with *N*-acetyl-l-glutamate kinase (NAGK) [5]. Furthermore, the NAGK binding sites of HpPII were conserved with *C. reinhardtii* and *C. variabilis* (Figure 4). Hence, HpPII likely behaves as a glutamine sensor through the C-terminal Q loop extension to control the activity of NAGK, and further to regulate nitrogen metabolism. The residues involved in ATP and 2KG binding sites were conserved as well. These results demonstrate that HpPII possibly acts as a signaling protein in nitrogen metabolism. However, a future study is needed to further investigate the contribution of HpPII in nitrogen regulation, targeting the astaxanthin biosynthesis. 

The expression of the PII-coding gene was species-specific between different phylogenetic clusters. For example, in cyanobacteria, the expression of PII-coding *glnB* was increased by nitrogen-poor conditions [31]. However, in the higher plant *Arabidopsis thaliana*, *GLB1* expression was not significantly affected by nitrogen [32]. It is worth noting that within the same phylogenetic cluster the *GLB1* expression to nitrogen depletion seems species-specific. The expression of *GLB1* in *H. pluvialis* (Hp*GLB1*) was enhanced under a nitrogen starvation condition. A similar result was also reported on the *GLB1* expression of green microalga *C. reinhardtii* [15]. The result in this study showed that Hp*GLB1* expression was stable in the first 24 h of nitrogen starvation, but increased dramatically at 48 h under nitrogen starvation condition. *C. reinhardtii GLB1* was induced from 0.5 h to 4 h upon nitrogen starvation [15]. In contrast, *C. variabilis’ GLB1* expression was independent of nitrogen availability [14]. As these microalgae can produce different secondary metabolic bioproducts upon nitrogen depletion, it is deduced that the *GLB1* expression is associated with different metabolic activities in microalgae. 

In this study, the astaxanthin content is correlated to the Hp*GLB1* expression. Similar to the Hp*GLB1* expression, the astaxanthin content was maintained at a low level within the first 24 h. The similar delayed astaxanthin increment was also observed previously in other *H. pluvialis* strains when exposed to stress condition [20]. In terms of the stable Hp*GLB1* expression, this may be associated with the initial physiological condition of cells, as these cells reaching exponential stage likely were exposed to N deficient condition prior to the N starvation test in this study. However, both of Hp*GLB1* expression and astaxanthin content started to increase at 48 h. Then, the astaxanthin content continuously increased after 48 h, even though it was under low light intensity (20 μmol m^−2^ s^−1^). It seems that the upregulation of Hp*GLB1* expression is likely regulated at a posttranslational level. Also, this is elucidated that there may be some physiological connection/interaction between nitrogen metabolism regulation and astaxanthin synthesis on *H. pluvialis* cells. A number of previous literatures have already confirmed that nitrogen condition plays an important role in *H. pluvialis* cells’ transformation, growth, and lipid and astaxanthin sythesis; the derived sequence of Hp*GLB1* and the characterization of HpPII in this study will facilitate further understanding on these metabolic pathways at the molecular level. More importantly, our results provide solid and credible bases for HpPII protein study. These will also provide new insight into the HpPII protein and its potential functions on the nitrogen-driven metabolic mechanism for astaxanthin production on *H. pluvialis*.

## 4. Materials and Methods

### 4.1. Microalga Strain and Culture Conditions

A strain of microalga *H. pluvialis* HPH was obtained from the Algae Collection at the College of Ocean and Earth Sciences in Xiamen University, China. The algal cells were cultivated in a 1-L glass vessel (15.5 cm in length and 9.5 cm in diameter) with Bold’s Basal Medium (BBM) [33] at 25 °C. The culture was continuously exposed to 20 μmol m^−2^ s^−1^ of light radiation, and 2.5% CO_2_ of aeration at a flow rate of 0.2 vvm [34]. When reaching the late exponential growth phase (approx. 5 × 10^5^ cells mL^−1^), the *H. pluvialis* cells were centrifuge-collected at 8000× *g* for 5 min. The cells were rinsed twice with nitrogen-free medium (BBM-N) and resuspended in BBM-N at a density of 5 × 10^5^ cells mL^−1^. The culture was continued for the trial (with triplicates), under the same cultivation condition. The culture was sampled with 50 mL (*n* = 3) at 0, 2, 4, 6, 8, 24, and 48 h, respectively. The sampled *H. pluvialis* cells (3 × 7) were collected via centrifugation and used for the subsequent RNA isolation, cDNA synthesis, and transcription analysis. 

### 4.2. RNA Isolation and cDNA Synthesis

The collected cells were washed with phosphate-buffered saline buffer (137 mM NaCl, 2.7 mM KCl, 10 mM Na_2_HPO_4_, 1.8 mM KH_2_PO_4_, pH 7.4), re-centrifuged, and immediately frozen in the liquid nitrogen. The frozen biomass was ground into a fine powder using mortar and pestle, which was cold-maintained by adding more liquid nitrogen. 50 mg of algal powder was used for total RNA extraction, which was achieved by a TaKaRa MiniBEST Universal RNA Extraction Kit (Takara Bio Inc., Kusatsu, Japan). The cDNA first-strand synthesis was carried out using High Capacity cDNA Transcription Kits (Applied Biosystems, Foster, CA, USA) according to the manufacturer’s protocol.

### 4.3. Gene Cloning and Rapid Amplification of cDNA Ends (RACE)

Partial cDNA of the PII protein coding gene of *H. pluvialis* (Hp*GLB1*) was amplified by reverse transcription PCR (RT-PCR) using degenerate primers GLB1-de-F and GLB1-de-R (Table 1). The PCR conditions for amplification were set as a 5 min polymerase activation step at 94 °C followed by 30 cycles of denaturation at 94 °C for 30 s, annealing at 56 °C for 30 s, extension at 72 °C for 1 min, and a final 10 min of extension at 72 °C. The PCR product was purified with an E.Z.N.A^®^ Gel Extraction Kit (Omega Bio-tek, Norcross, GA, USA), cloned into a pMD^TM^19-T Vector (Takara Bio Inc., Kusatsu, Japan), and then sequenced at the Invitrogen Corporation (Waltham, MA, USA).

In order to generate the full length cDNA of the Hp*GLB1*, this study used the SMART™ RACE cDNA Amplification Kit (Clontech, Mountain View, CA, USA) to perform 3′ and 5′ rapid amplification of the cDNA ends (RACE). Based on the obtained PCR results, the primers GSP1 and GSP2 (Table 1) were designed for the 5′ RACE and 3′ RACE. Subsequently, genomic DNA of *H. pluvialis* was isolated using the cetyltrimethylammounium bromide (CTAB) method [35]. Primers GLB1-F and GLB1-R were used to amplify genomic DNA of Hp*GLB1*. As presented, all the PCR products were purified, cloned, and sequenced.

### 4.4. Bioinformatics Analysis

The open reading frame (ORF) was determined by BioEdit software. The deduced amino acid sequence was analyzed with Primer Premier 5. The homology searches for nucleotide and amino acid sequence similarities were conducted with Clustal W and Blast (http://www.ncbi.nlm.nih.gov/). The phylogenetic tree was constructed according to the amino acid sequences of the PII signaling protein by MEGA 6.06 software. Theoretical isoelectric point (pI) and molecular weight (Mw) were calculated with Compute pI/Mw tool (http://web.expasy.org/compute_pi/).

### 4.5. Plasmid Construction

Based on the nucleotide sequences of start and stop codons of the Hp*GLB1* gene, the gene specific primers GLB1-B-F and GLB1-B-R were used to amplify the full length of the coding region. The PCR performance was programmed as 94 °C for 5 min followed by 30 cycles of denaturation at 94 °C for 30 s, annealing at 65 °C for 30 s, extension at 72 °C for 1 min, and a final 10 min extension at 72 °C. The 5′ and 3′ ends of amplified Hp*GLB1* coding region contain a Bam*H*I and an Eco*R*I restriction site, respectively. The amplified coding region was digested with Bam*H*I and Eco*R*I restriction endonucleases and inserted into the corresponding sites of pET28a for generating the recombinant plasmid pET28a-HpG*LB1*, and then introduced into *Escherichia coli* BL21 (DE3) for expression. 

### 4.6. Expression and Purification of HpPII

HpPII protein expression and purification were performed according to the previous method [36], with some modifications. Briefly, the *E. coli* BL21 (DE3) harboring pET28a-HpG*LB1* was cultured in 10 mL of Luria-Bertani (LB) broth with 50 μg mL^−1^ of kanamycin (sigma-Aldrich, St. Louis, MO, USA) at 37 °C and retained on a shaker (200 rpm) overnight. Then, 0.5 mL of the resulting culture was inoculated to 50 mL of LB broth supplemented with 50 μg mL^−1^ kanamycin and incubated under the same condition. When the bacterial concentration reached 0.6 of OD_600_ value, the culture was added with 0.1 mM isopropyl β-D-1-thiogalactopyranoside (IPTG) and incubated for another 6 h. The cells were then harvested by centrifugation (4 °C, 5000× *g*, 10 min) and washed twice with sodium phosphate buffer (200 mM Na_3_PO_4_, 300 mM NaCl, pH 7.4). The pellets were suspended in the sodium phosphate buffer and lysed by sonication (100 W, 60 cycles of 5 s sonication), followed by 5 s incubation on ice. Finally, the soluble lysate was centrifuged (4 °C, 10,000× *g*, 10 min) for collection. The HpPII protein was purified with Ni-NTA His•Bind Resin. The molecular mass and purity, plus the total proteins, were checked by 12% SDS-PAGE.

### 4.7. Expression Analysis of HpGLB1 by qRT-PCR

A total of 21 cDNA samples derived from the control and nitrogen depleted treatments (triplicates sampling at 0, 2, 4, 6, 8, 24, and 48 h) were used to determine mRNA expressions by real-time quantitative reverse transcriptase PCR (qRT-PCR). The initial samples (0 h) were treated as the control. qRT-PCR was performed on an ABI-7000 System (Applied Biosystems, Foster, CA, USA) using iTaq^TM^ Universal SYBR^®^ Green Supermix (Bio-Rad, Hercules, CA, USA). Primers GLB1-Q-F and GLB1-Q-R were designed to amplify the 120 bp sequence of HpPII for qRT-PCR. The 18S ribosomal RNA gene expression was used as the internal control. Each 20 μL PCR reaction consisted of 10 μL iTaq^TM^ Universal SYBR^®^ Green Supermix, 1 μL of forward and reverse primers (10 μM) each, and 8 μL of cDNA. The thermal cycles were set as stage 1: 95 °C for 10 min; stage 2: 40 cycles of 95 °C for 15 s, and 60 °C for 1 min; and stage 3: 1 cycle of 95 °C for 15 s, 60 °C for 1 min, and 95 °C for 15 s. The qRT-PCR data were analyzed by 2^−ΔΔCT^ method based on the cycle threshold (Ct) values. 

### 4.8. Astaxanthin Extraction and Quantification

The freeze-dried cells (10 mg, taken at 0, 24, 48, 72, and 96 h with triplicated) were mixed with 0.5 g glass beads and vortexed for 2 min. Then 5 mL dichloromethane/methanol (1:1, *v*/*v*) was added and vortexed for 1 min. The mixture was centrifuged at 3000× *g* at 4 °C for 3 min, and the supernatant was collected. The pellet was extracted repeatedly with 3 ml dichloromethane/methanol (1:1, *v*/*v*) until it became colorless. The combined supernatant was dried in nitrogen gas, reconstituted with 1 mL of 0.025 M NaOH, and then saponified at 4 °C for 4 h. The pigment extract was diluted with 1 mL of the mix of methanol and acetonitrile/methanol (3:1), and used for astaxanthin quantification.

Separation and identification of astaxanthin was carried out on an Acquity UHPLC system (Waters) equipped with photodiode array (PDA) detector. Samples (5.0 μL) were quantitatively injected into an Acquity UPLC Shield C18 BEH column (2.1 × 100 mm, 1.7 μm particle size; Waters, Milford, WI, USA). The eluents were (A) 50% (*v*/*v*) acetonitrile in demineralized water; (B) acetonitrile; and (C) ethyl acetate, which all contained 0.10% (*v*/*v*) formic acid. The flow rate was maintained at 300 μL min^−1^. The program was initiated from 25% A/75% B and then as follows: 0–10 min—linear gradient to 100% B, 10–15 min—isocratic at 100% B, 15–20 min—linear gradient to 87.5% B/12.5% C, 20–21 min—linear gradient to 70% B/30% C, 21–28 min—linear gradient to 100% C, and 28–29 min—isocratic at 100% C. After 29 min, the eluent composition reverted to its initial composition in 1 min followed by an equilibration phase of 12 min. Detection wavelength for UV–vis was adjusted to 460 nm. Since astaxanthin esters were saponified, the free astaxanthin peak represented total astaxanthin. The astaxanthin peak was identified by comparing retention time and spectra with astaxanthin standard (Sigma-Aldrich, St. Louis, MO, USA). The quantification of astaxanthin was determined by a calibration curve obtained with astaxanthin standard.

### 4.9. Statistical Analysis

The experiment was conducted with biological triplicates, and the data measurement was performed in triplicates. The data in the figure were showed as mean ± SE in this study. The statistical differences of transcription level between sampling times were detected by one-way ANOVA analysis using IBM SPSS Statistics 24, with nitrogen starvation time as variance and relative transcription level as dependent variables, followed by Duncan’s test with a significant level of 0.05.

## Figures and Tables

**Table 1:** Primer sequences used in this study.

Name	Sequence (5′–3′)	Sequence Information
GLB1-de-F	AGCGNTACGCN GGCACNGAGTT	Homology cloning primer
GLB1-de-R	ATGTCCTCCATGCCNCCCTCCAT	Homology cloning primer
GSP1	GATTACGCCAAGCTTACTATGTCCACCTGGGAGCGTGAC	3’-RACE primer
GSP2	GATTACGCCAAGCTTCGCTCCCAGGTGGACATAGTGAC	5’-RACE primer
GLB1-F	ATGATTGGCACAGTGCTGCAGAA	Gene cloning primer
GLB1-R	CTAACCACTAGCCTCCGGCTTG	Gene cloning primer
GLB1-B-F	GGAATTCATGATTGGCACAGTGCTGCAGAA	Gene cloning primer
GLB1- B-R	CCCAAGCTTCTAACCACTAGCCTCCGGCTTG	Gene cloning primer
GLB1-Q-F	CGCCTGGCATTTGTCATTG	Real-time gene primer
GLB1-Q-R	AAACTCAGTGCCAGCATAGCG	Real-time gene primer
18S-F	CAAAGCAAGCCTACGCTCT	Real-time gene primer
18S-R	ATACGAATGCCCCCGACT	Real-time gene primer
